# Beyond a spec: assessing heterogeneity in the unregulated opioid supply

**DOI:** 10.1186/s12954-024-00980-5

**Published:** 2024-03-15

**Authors:** Lea Gozdzialski, Rebecca Louw, Collin Kielty, Ava Margolese, Eric Poarch, Miriam Sherman, Fred Cameron, Chris Gill, Bruce Wallace, Dennis Hore

**Affiliations:** 1 Department of Chemistry, University of Victoria, Victoria, V8W 2Y2 Canada; 2 School of Social Work, University of Victoria, Victoria, V8W 2Y2 Canada; 3 Canadian Institute for Substance Use Research, University of Victoria, Victoria, V8N 5M8 Canada; 4 Department of Computer Science, University of Victoria, Victoria, V8W 3P6 Canada; 5 SOLID Outreach, Victoria, V8T 1C6 Canada; 6 Applied Environmental Research Laboratories (AERL), Department of Chemistry, Vancouver Island University, Nanaimo, V9R 5S5 Canada; 7 Department of Chemistry, Simon Fraser University, Burnaby, V5A 1S6 Canada; 8 Department of Environmental and Occupational Health Sciences, University of Washington, Seattle, 98195 USA

**Keywords:** Drug checking, Opioids, Quantification, Heterogeneity, Paper spray mass spectrometry

## Abstract

**Background:**

Drug checking services aim to provide compositional information for the illicit drug supply and are being employed in public health responses to extreme rates of overdose associated with fentanyl within street opioids. The technologies used within these services range from basic qualitative tests, such as immunoassay test strips, to comprehensive quantitative analyses, such as mass spectrometry. In general, there is concern that heterogeneity of a drug mixture adds significant uncertainty when using drug checking results based on a small subsamples. The presence of hot spots of active drug components in this context is often termed the ‘chocolate chip cookie effect’. Establishing the limitations of the service are essential for interpretation of the results.

**Methods:**

This study assesses the consequence of drug heterogeneity and sampling of consumer level opioid purchased in Victoria, British Columbia ($n=21$, 50–100 mg each) on quantitative fentanyl results determined from testing with paper spray mass spectrometry.

**Results:**

Using descriptive statistics, such as relative standard deviation and interquartile range, the results demonstrate varied distributions of fentanyl concentrations within a single drug batch. However, the presence of hot spots, defined as outliers, were relatively rare.

**Conclusions:**

This study found that the variability in fentanyl concentration from drug heterogeneity and sampling is greater than that attributed to the analytical technique. On a practical level, this provides data to help guide communication of limitations of drug checking services, supporting the aim of trust and transparency between services and people who use drugs. However, if drug checking services continue to be restricted from fully engaging with the reality of manufacturing, buying, selling, mixing and dosing practices, the accuracy, usefulness, and impact will always be limited.

**Supplementary Information:**

The online version contains supplementary material available at 10.1186/s12954-024-00980-5.

## Introduction

Drug checking is a crucial tool in harm reduction because it provides information about the composition of substances to people who use drugs [1, 2]. Depending on the technology used at a service, this information might be qualitative and at a variable level of specificity. Fentanyl immunoassay test strips are a method employed in drug checking for the broad detection of fentanyl and fentanyl analogues [3–10]. This drug checking method does not provide a measure of concentration nor the specific identity of the fentanyl analogue. Even so, early literature reports positive perspectives on fentanyl immunoassay test strips from people who use drugs, particularly for those who are not wanting to consume opioids or more specifically, fentanyl [4]. In such cases, a positive test often prompted a positive risk reduction behaviour change such as ensuring naloxone is available prior to consumption, using with a friend, or even discarding the drug [3, 4, 10]. While extremely useful for testing of non-opioids, overdose reduction is unlikely as a direct result of fentanyl test strip use in cases where fentanyl is expected. However, value is found in the binary test for a range of related reasons. In some markets, where the opioid market is assumed to predominately contain fentanyl, people report using fentanyl test strips to either confirm that presumption and curiosity, or inform harm reduction practices. In some cases, people use the strips to negotiate with dealers and ensure fentanyl was in fact present in their purchase [6]. Ultimately, relying on the fentanyl test strip alone had limited usefulness in such a complex drug market [6].

The abilities of drug checking technologies are getting more accurate and comprehensive with time as a result of the drug checking community and researchers adapting analytical tools to best respond to the complex opioids most often linked to overdose. The call for more in-depth compositional information has motivated drug checking services using spectroscopy or other analytical instrumentation. Concurrently, the limitations and capabilities of these techniques for drug checking have been identified through ongoing practice. For instance, many services in North America are moving beyond binary (e.g. fentanyl test strips) to qualitative testing of expected opioids. For example, informing someone their sample contains fentanyl and caffeine identifies the substances within the sample. This information offers additional information to the test strips, including the identity of cutting agents and in some cases other drug components. In an ideal case, however, drug checking technologies, such as paperspray mass spectrometry (PSMS), provide information that also offers quantitative information. People who use drugs have identified such quantitative information as important for the usefulness of drug checking [11]. Accurate quantitative information is valuable to navigate the significant variability in the unregulated drug market [12–14] and inform dosing to achieve the desired experience and reduce overdose risks [11, 15]. For example, informing someone their sample contains 5% fentanyl and 95% caffeine. Someone who consistently uses drug checking services might know that this is approximately the strength they are used to. Such quantitative information has also been noted as especially desired for people selling or sharing drugs, to prevent misrepresenting samples [16, 17]. Some service providers have even expressed hesitancy in implementing drug checking technologies unless they provide both high accuracy and quantitative results [18]. Accurate quantification information is also valued for public health monitoring and reporting of the illicit market and valued as amongst overdose prevention strategies [19]. Evaluating whether the accuracy of a technology is acceptable for such purposes becomes especially challenging, recognizing that accuracy demonstrated under laboratory conditions might not hold when applied point-of-care to the unpredictable drug market [18, 20].

Beyond the accuracy of a method, sampling procedures are far less evaluated and discussed in the context of drug checking. Typically, less than 5 mg of a drug sample is required for testing, depending on the instrumentation or analytical method. The limited quantity is an attractive feature of the service because people do not have to sacrifice a large amount of their personal supply, lowering one barrier to using drug checking services routinely [11]. However, the homogeneity of a drug sample, i.e. how uniformly mixed the active compounds and cutting agents are, is a common concern in the discussion of the limitations and risks of drug checking [21, 22]. Among people who support, facilitate, and use drug checking, the topic of heterogeneity is informally referred to as the “chocolate chip cookie effect” [23]. In this definition, the chocolate chips represent the active drug compound distributed within a sample (the cookie). In other words, is testing a subsample of a drug mixture truly representative of the larger quantity that constitutes a personal dose? At the extreme case, a poorly mixed batch might have subsamples that consist of the active compounds or adulterants (chocolate chips), rather than represent the drug sample itself. Alternatively, the subsample might miss the active compounds or under-represent the proportion of active drug components in the larger sample. This potential of “hot spots” complicates quantitative drug checking protocols and informed dosing, especially for potent opioids such as fentanyl. In general, the growing body of drug checking protocols mention that it is essential to ensure that a sample submitted to drug checking is homogeneous and representative for accurate quantification of substances [24]. Notably, while there are established mixing practices and methods to reduce heterogeneity, there are limited ways to ensure this is happening at a consumer level and when a subsample is submitted at a drug checking service this is mostly unknown. Answering the question about whether a drug mixture in powder form is homogeneous depends on the scale in question. This quantity is termed the scale of scrutiny [25]. For example, if a larger batch of 10 g is divided into individual doses of 100 mg, the objective is that each dose contains the same amount of active drug. Here, the scale of scrutiny would be 100 mg. One of the biggest challenges is when characterization methods, like the instruments used in drug checking, are not appropriate for the scale of scrutiny (i.e. sample size used is too large or too small). Most drug checking methods require only a small amount of drug sample, which in most cases is significantly below the quantity of a dose.

Despite ongoing speculations of the prevalence of the “chocolate chip cookie effect” as a limitation of drug checking, no formal study on this topic exists in the context of drug checking and specifically the opioid supply currently linked to unprecedented rates of overdose. The importance of transparency surrounding technical limitations is emphasized in nearly all evaluations of drug checking [26]. It would be an oversight, now, to ignore the challenges faced by heterogeneity, within the context of aiming to create a more regulated and consistent supply. For example, how much of a limitation is the heterogeneity of drug samples in drug checking results? Based on the ongoing assumption that the chocolate chip effect is a limitation of drug checking, it is then not straightforward to address this challenge in terms of procedural steps or messaging considerations. This study aims to assess the heterogeneity of a typical personal supply of opioids at the consumer-level and provide implications for practice and policy. In doing so we seek to determine the risks associated with the “chocolate chip cookie” effect and comment on considerations when extrapolating typical drug checking results from a small 5 mg sample to a larger personal supply. We provide a practice guideline for drug checking to minimize the impact of heterogeneity on drug checking results and clearly communicate quantitative results.

## Methods

### Sample acquisition

**Fig. 1:**
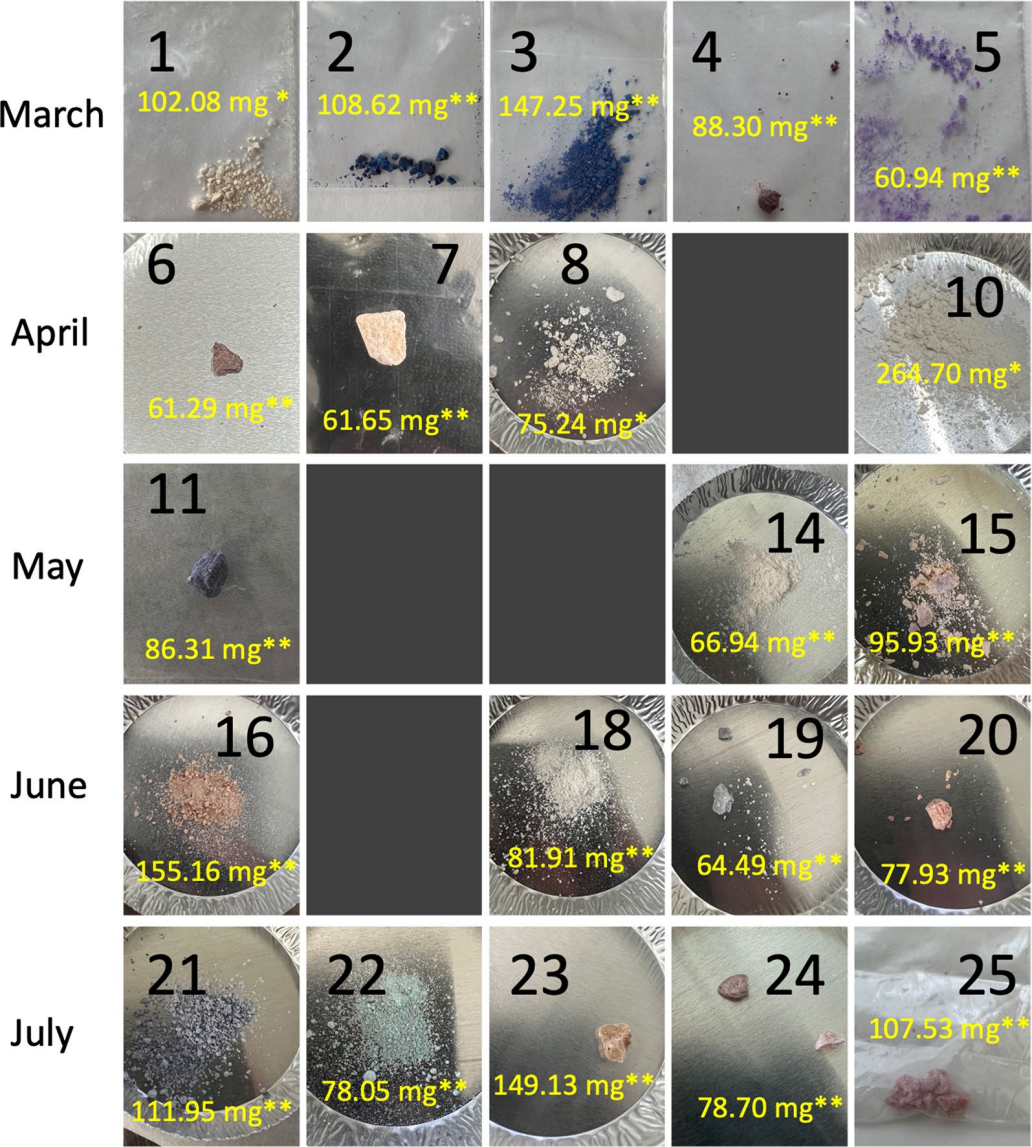
Photos of the batches collected prior to separation. Five batches were collected each month from March 2022 to July 2022. Greyed out boxes represent batches that were excluded from the analysis presented here. The total weight is presented for each batch. The expected concentrations based on pre-testing intake questions are indicated by ($$*$$) for “low-level street down” and ($$**$$) for “strong down”

**Fig. 2:**
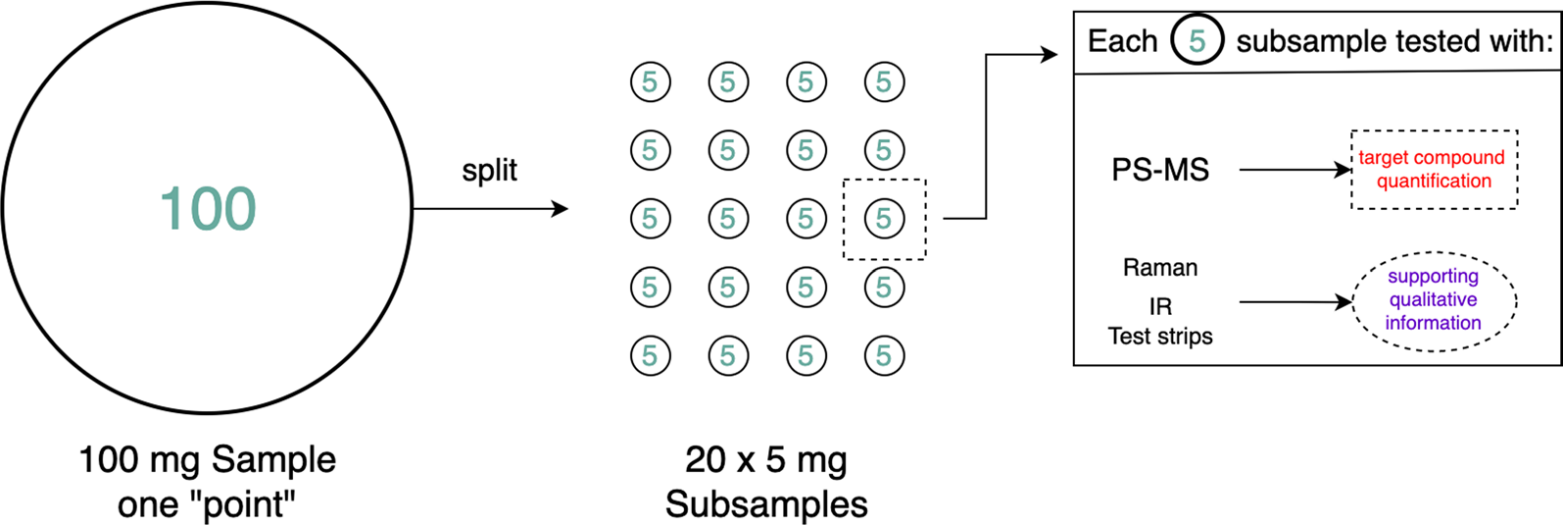
Flow diagram of the sampling procedure for each batch investigated in the study. Each subsample ($n=$10–20, 5 mg each) was tested using the suite of instruments used in the drug checking service. The distribution of results for the subsamples was evaluated

Several samples ($n=25$) ranging from 50 to 100 mg, referred to here as batches, were submitted to the drug checking service, shown in Fig. 1. These batches were collected over the four month period of May 2022 to July 2022, with five batches collected each month. Each batch was separated into 10–20 subsamples (about 5 mg each, a typical quantity of drug received at the drug checking service). Each subsample was crushed and mixed in an Eppendorf tube with a spatula and prepared for PSMS analysis. The sampling process is outlined in Fig. 2. Variability in concentration of fentanyl through the sample was quantitatively assessed from the PS-MS data. The PSMS method has been detailed in several recent publications [27–30]. Four batches were excluded from the final analysis where the quantity provided was less than the intended amount (< 50 mg) or where the batch was found to be fentanyl at a concentration greater than 80% by weight (hereafter referred to as w/w%) and no cutting agents were detected. Sample intake questions and consent were recorded as described in the corresponding ethics protocol (REB H20-03384), that included information such as what the sample is supposed to be and any unexpected effects.

### Descriptive statistics

Descriptive statistics were used to describe the variability in each batch. The coefficient of variation (CV), also known as relative standard deviation (RSD), is the common metric used to describe the heterogeneity of powders and pills. The coefficient of variation is defined as the standard deviation of the concentration between samples divided by the average concentration ($\sigma /{\bar{\mu }}$). Interquartile range (IQR) was also used to discuss the heterogeneity on an approximate 5 mg scale. We consider the presence of outliers, i.e. subsamples where the fentanyl concentration extends beyond the 1.5 times the interquartile range, as an indication of “hot spots”. Analytical error was estimated through repeat acquisitions ($n=5$) of the same sample for the first and last subsample on each batch.

## Results

**Fig. 3:**
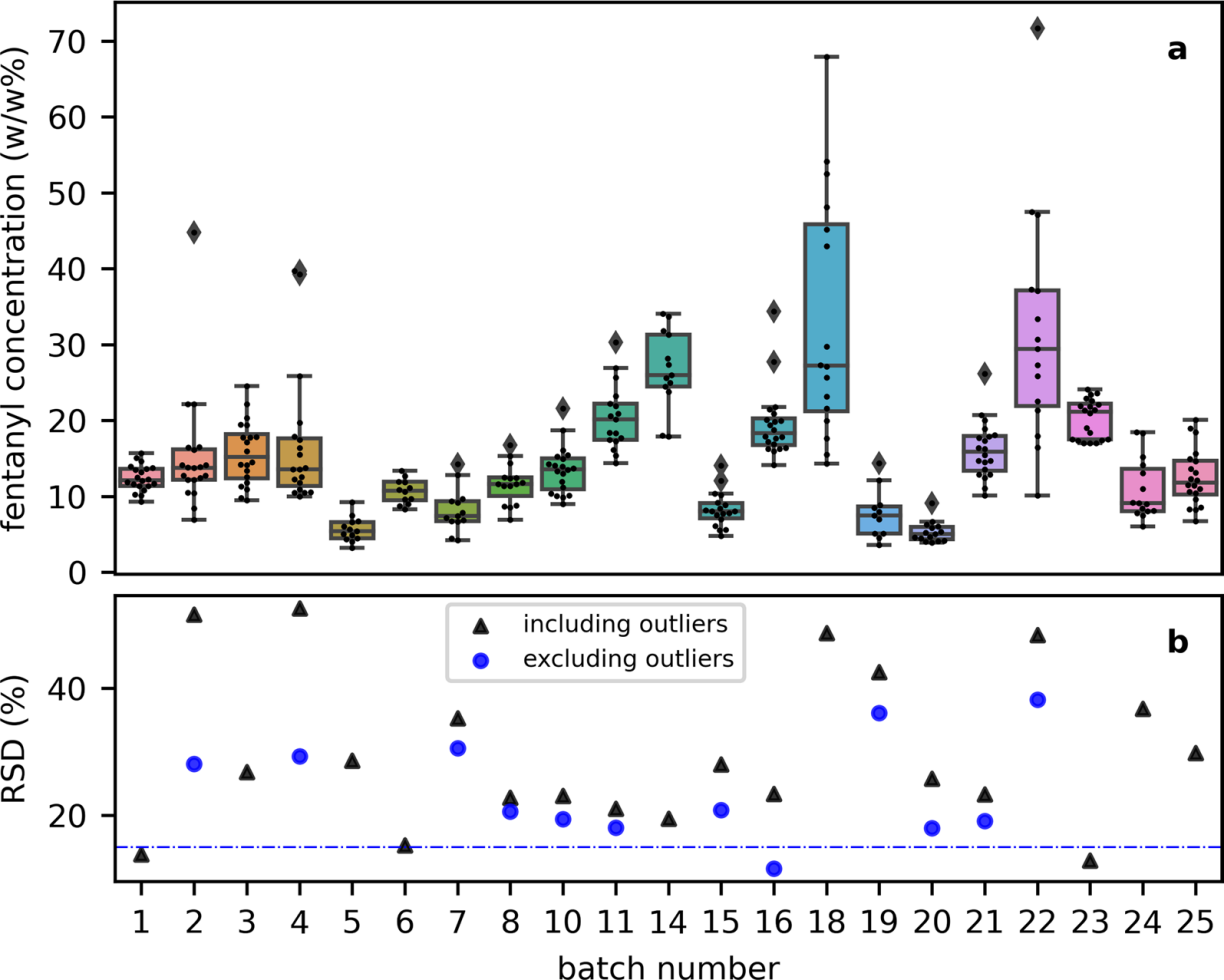
The distribution of fentanyl concentration (w/w%) for each sample batch. Each point represents the result for an individual subsample, with the box-and-whiskers plot summarizing the distribution. The median (q50) is the horizontal line within the box. The bounds of the box represent the IQR (q25–q75), and the whiskers extend to 1.5$\times$IQR. Outliers are identified as outside these bounds

A box and whiskers plot of the distribution of fentanyl concentration in each batch, as determined by PSMS, is shown in Fig. 3a. In the more extreme case, drug checking results for multiple subsamples ($n=15$) from one batch (#18) provided fentanyl quantification results ranging from 10 to 72 w/w%. In another case (#20), quantification results had a much narrower range, spanning 5 w/w% (e.g. 4–9 w/w%). Outliers were identified in several batches. Of 352 subsamples included in this study, 15 were identified as outliers (approximately 5% of subsamples). Of note, the outliers identified were exclusively on the upper end and no samples were found to contain only cutting agents. A distribution around the mean was a more common observation. For a majority of the batches the IQR ranged from 1.6$-$6.9 w/w%, with two batches having broader distributions (IQR >15 w/w%). The data for fifteen batches showed an approximate normal distribution (Additional file 1: Table S1), as calculated using Shapiro-Wilk normality test [31]. Since the sample size here is very small we caution the over-interpretation of this result, however it does suggest some uniformity within the powder mixtures at a 5 mg scale.

The RSD for the batches were calculated for the dataset inclusive and exclusive of the identified outliers and shown in Fig. 3b. The cumulative probability of the RSD for fentanyl concentration in a “typical” opioid batch is shown in Additional file 1: Figure S1, with an average measurement RSD calculated as 25%.

Numerical data represented in Fig. 3 are additionally summarized in Additional file 1: Table S1 in the Supplementary Information. The mean, IQR, and RSD of repeated acquisitions of the select subsamples are shown in Additional file 1: Table S2, as an indicator of analytical variability. The average analytical RSD was calculated as 5%. Therefore, where $S^2 = S^2_{\textrm{analytical}} + S^2_{\textrm{sampling}} + S^2_{\textrm{mixing}}$, most variation in analysis is attributed to mixing and sampling for the current PSMS procedure.

## Discussion

The “chocolate chip cookie effect” has been an assumed limitation of drug checking services by drug checking service providers and users alike, notably for the testing and reporting of fentanyl samples in which possible “hot spots” may result in inaccurate reporting of the overall sample [23, 32]. This research sought to test the validity of the assumed “chocolate chip cookie effect” through an examination of typical fentanyl samples provided to the drug checking project for research purposes. This is, to the best of our knowledge, the first research-based demonstration of the impact of drug heterogeneity of a typical consumer-level fentanyl sample on drug checking results. Community drug checking is considered in many ways an evidence-making intervention more than an evidence-based intervention [33, 34]. In this way, this collaboration between drug checking providers and people who use drugs bridges community observations to analytical answers about the risks of heterogeneity in the drug supply. Such co-creation of knowledge best informs the implementation of drug checking practices as well as new interventions needed to mitigate these risks [26, 33, 35].

Overall, this study validates the “chocolate chip cookie effect” as an inherent risk of drug checking when subsampling street samples. The methods used for mixing the sample, the inherent chemical properties of powders (varied particle sizes, densities, and chemical interactions between molecules), and bias in sampling contribute to this uncertainty [36–39]. These results confirm that drug heterogeneity will affect the precision of quantitative results and this limitation needs to be communicated to service users, however, beyond the simple “chocolate chip cookie” analogy. We document two potential manifestations of the “chocolate chip cookie effect”. While rare, heterogeneity can be extreme in poorly mixed samples. The more typical presentation of fentanyl heterogeneity is a distribution around the mean concentration, indicating both a level of variability but within a degree of uniformity. We do not attempt to calculate the degree of acceptable variability in fentanyl concentrations for personal use, which we expect is unknown and depends on the person and drug use practices.

### Communicating limitations

Based on these results, we see opportunities to enhance how to communicate sampling and heterogeneity beyond the “chocolate chip cookie effect” that extends information to service users on how to assess and respond to this limitation. The findings from this single study could potentially offer the following assessment of the limitation and risks:

- Even with quantitative testing, directly reporting precise results to individuals currently excludes uncertainty due to heterogeneity risks.
- The majority of current public health messaging around the “chocolate chip cookie” effect focuses almost exclusively on the risk of hot spots, in the context of fentanyl test strips possibly missing the detection of fentanyl due to heterogeneity [32]. This limited messaging ignores populations that access drug checking to quantify substances like fentanyl that are heavily diluted due to their high potency.
- On an individual level, there are inherent limitations in quantifying the uncertainty given a single drug check. While the average RSD for this study was found to be around 25%, it ranged from 15 to 53% for individual batches. Lack of standard drug mixing and manufacturing practices contribute to this variability.
- Service users should expect quantitative drug checking results to represent a value within the distribution around the average concentration of their sample. For instance, a result of 12 w/w% verses 13 w/w% may not be significant, and the average of such sample might actually be closer to 14 w/w% if tested on a greater scale.
- Harm reduction messaging needs to further emphasize the value of mixing for improved safety in drug checking, drug dosing, and drug consumption. Homogenizing (extra mixing, grinding) larger quantities of a batch will result in more representative drug checking results.

Effective communication of drug checking results and limitations is essential not just to inform service users’ actions but also to develop and maintain community trust in the accuracy of the service. A recent review of drug checking literature identified the gaps and challenges in assessing outcomes from drug checking services, in particular, best practices for messaging and communication strategies [40]. Building new ways to communicate heterogeneity can follow the recommendations of others for meaningful engagement in the development of messaging [41–43]. Evaluations of how people interpret and navigate risks, based on what can be confirmed and what is left unknown, will enhance drug checking effectiveness [44–47].

### Practices to reduce risks associated with sample heterogeneity

While increasing the sample size and/or number of samples from a product could address or minimize heterogeneity [48–50] we do not perceive this to be the most relevant option for most services testing fentanyl and other illicit drugs. Requiring an entire dose or more would make drug checking unrealistic and inaccessible to most for personal, judicial, and financial reasons. Criminalization and safety concerns make carrying bulk substances into a drug checking service high risk for people who use drugs and people who sell drugs [34, 51, 52]. In a recent assessment of police perception on drug checking service in Scotland, for instance, it was emphasized that it would be very hard to say with complete assurance that someone would not be criminalised in such cases [53]. In numerous studies it has also been identified that the small amount of drug required is an attractive and barrier-lowering feature of drug checking services [11].

Current practices to address heterogeneity exist namely encouraging thorough mixing of a sample pre-testing and these can continue to be emphasized in standard operating practices. For homogeneity of illicit drug mixtures to be achieved on a milligram scale the particle size must be significantly reduced (i.e. finely ground and homogenized) [54]. However, providing the resources for people to access proper mixing equipment within a point-of-care setting seems to be challenging and unexplored to date.

### Directly addressing the heterogeneity problem

Fentanyl mixtures are increasingly complex with the potential of differing fentanyl analogues present along with other notable activities such as xylazine, nitazenes or a benzodiazepine along with cutting agents. The overdose crisis related to these substances is also a complex problem and without simple solutions. Our findings uncover a limitation of drug checking as an overdose response that cannot be solved by more accurate technology. We see value in questioning the unique potential for drug checking to expand its mandate beyond testing and reporting the risks, to new practices to reduce the risks of the heterogeneous, complex and potent supply most linked to overdose and harms [55, 56].

Heterogeneity in illicit drugs can be addressed, not only reported. If the problem is the heterogeneity of street fentanyl how can public health improve homogeneity to reduce the risk of overdose and harms? Rather than viewing drug checking as an intervention that informs people who use drugs to change behaviors, what are the ways drug checking can intervene to change the drug supply recognized to be a root cause of the overdose crisis? [55–57] We view drug checking to have untapped potential by public health due to precautionary approaches that avoid engagement with the illicit drug market that we are testing [52, 57, 58]. We recommend exploring practices such as providing information and guidance on established pharmaceutical mixing techniques, as well as the necessary supplies for proper mixing including buff/diluent to reduce concentrations to a potentially safer or anticipated level. Such interventions are typically rejected as contributing to the process of drug production and selling rather than an upstream action to reduce risks through market interventions [57, 58].

### Limitations

As these results are based on a small subset of samples in Victoria, British Columbia, this study does not comment on the likelihood of the same distributions on a population level, although it does validate continued observations by people who use drugs and people who deliver drug checking services. Notably, this study reflects what was actively being done in our drug checking service for a typical drug check; i.e. we did not make any adjustments to the sampling protocol, which may include biases associated with random and convenience sampling when splitting the batches into subsamples. This sampling procedure might not be representative of other drug checking services.

## Conclusions

The “chocolate chip cookie effect” is an assumed limitation of current drug checking practices, notably when testing fentanyl mixture samples that are heavily diluted due to their potency. Establishing the actual limitations of this effect related to heterogeneity is necessary to maintain trust from service users and  confidence in public health reporting. A collaboration between the drug checking project and a drug user organization in Victoria, BC, Canada assessed the possible consequences of heterogeneity in consumer level opioid purchases over a four month period. The results confirm street fentanyl samples to exhibit heterogeneity linked to imperfectly mixed samples of powder fentanyl, resulting in a limitation for drug checking to accurately report on the overall sample. However, the “chocolate chip cookie” analogy may be an imperfect message as “hot spots” were rare. The risk of not detecting and reporting fentanyl was insignificant, while accurately quantifying the concentration of fentanyl with a single test proved to be challenging, as concentrations varied between subsamples. We found the typical presentation of fentanyl heterogeneity to be a distribution around the mean concentration, indicating both a level of variability but within a degree of uniformity. Where drug checking programs are reporting fentanyl concentrations, messaging of the expected range and possible uncertainly is necessary. Furthermore, harm reduction messaging on the value of mixing fentanyl products could reduce heterogeneity and improve safety. Heterogeneity of fentanyl concentrations within street opioids is one more risk faced by people who use drugs. Public health responses that reduce these risks require a willingness to more fully engage in the illicit drug market that we are testing and support interventions that enable access to supplies for proper mixing and dosing.

### Supplementary Information

**Additional file 1.** Summary of descriptive statistics by batch;  **Table S1.** Summary of descriptive statistics for repeat measurements;** Table S2.** Cumulative probability for the RSD of percent fentanyl; Figure S1.

## Data Availability

The datasets used and/or analysed during the current study are available from the corresponding author on reasonable request.

## Abbreviations

CV: Coefficient of variation
IQR: Interquartile range
PSMS: Paperspray mass spectrometry
RSD: Relative standard deviation
