# Supplementary material for: Beyond a spec: assessing heterogeneity in the unregulated opioid supply
Source: Harm Reduct J. 2024 Mar 15;21:63. doi: 10.1186/s12954-024-00980-5 (PMC10941387; doi:10.1186/s12954-024-00980-5)
Supplement: Supplementary file 1 — Additional file 1. Summary of descriptive statistics by batch; Table S1. Summary of descriptive statistics for repeat measurements; Table S2. Cumulative probability for the RSD of percent fentanyl; Figure S1. [file 12954_2024_980_MOESM1_ESM.pdf]

Supplementary Materials for

Beyond A Spec: Assessing Heterogeneity in the Unregulated Opioid Supply

Lea Gozdzialski[a], Rebecca Louw[b], Collin Kielty[a,b], Ava Margolese[a,b], Miriam Sherman[a,b], Eric Poarch[a,b], Fred Cameron[h], Chris G. Gill[c,a,b,d,e], Bruce Wallace[b,f], Dennis K. Hore[a,g]

[a]*Department of Chemistry, University of Victoria, Victoria, British Columbia, V8W 3V6, Canada*
[b]*Canadian Institute for Substance Use Research, University of Victoria, Victoria, British Columbia, V8W 2Y2, Canada*
[c]*Applied Environmental Research Laboratories (AERL), Department of Chemistry, Vancouver Island University, Nanaimo, British Columbia, V9R 5S5, Canada*
[d]*Department of Chemistry, Simon Fraser University, Burnaby, British Columbia, V5A 1S6, Canada*
[e]*Department of Environmental and Occupational Health Sciences, University of Washington, Seattle, Washington, 98195, United States*
[f]*School of Social Work, University of Victoria, Victoria, British Columbia, V8W 2Y2, Canada*
[g]*Department of Computer Science, University of Victoria, Victoria, British Columbia, V8W 3P6, Canada*
[h]*SOLID Outreach, Victoria, British Columbia, Canada*

Table S1: Descriptive statistics for fentanyl concentration for each drug batch, including the number of subsamples, mean, median, interquartile range (IQR), min and max concentration of the subsamples within a batch, number of outliers, and relative standard deviation (RSD).

batch num	mean (w/w%)	median (w/w%)	IQR (w/w%)	min value (w/w%)	max value (w/w%)	n outliers	RSD (%)	normal distr.
1 (n=20)	12.4	12.1	2.3	9.3	15.7	0	16	True
2 (n=20)	15.3	13.8	4.0	6.9	44.8	1	52	False
3 (n=20)	15.7	15.2	5.8	9.5	24.5	0	25	True
4 (n=19)	16.9	13.6	6.3	10.0	39.7	2	53	False
5 (n=13)	5.6	5.4	2.1	3.2	9.2	0	36	True
6 (n=12)	10.6	10.7	2.4	8.3	13.4	0	19	True
7 (n=13)	8.2	7.4	2.6	4.2	14.2	1	37	True
8 (n=15)	11.6	11.6	2.4	6.9	16.7	1	26	True
10 (n=20)	13.5	13.6	4.0	9.0	21.6	1	22	True
11 (n=17)	20.4	20.2	4.8	14.4	30.3	1	20	True
14 (n=13)	26.7	26.0	6.9	17.9	34.1	0	19	True
15 (n=18)	8.3	8.0	2.0	4.8	14.1	2	24	True
16 (n=20)	19.5	18.3	3.5	14.1	34.4	2	26	False
18 (n=16)	33.3	27.2	24.7	14.3	67.9	0	48	True
19 (n=11)	7.7	7.4	3.6	3.6	14.4	1	39	True
20 (n=15)	5.3	5.0	1.6	3.9	9.1	1	19	False
21 (n=20)	16.1	15.8	4.5	10.1	26.1	1	25	True
22 (n=15)	31.7	29.4	15.2	10.1	71.7	1	47	True
23 (n=20)	20.2	21.1	4.7	17.0	24.1	0	15	False
24 (n=15)	10.9	9.1	5.6	6.0	18.5	0	37	False
25 (n=20)	12.5	11.8	4.5	6.7	20.1	0	32	True

A cumulative probability graph is shown in Fig S1. Given the current dataset such calculation can indicate expected levels of variation in fentanyl concentration within a batch.

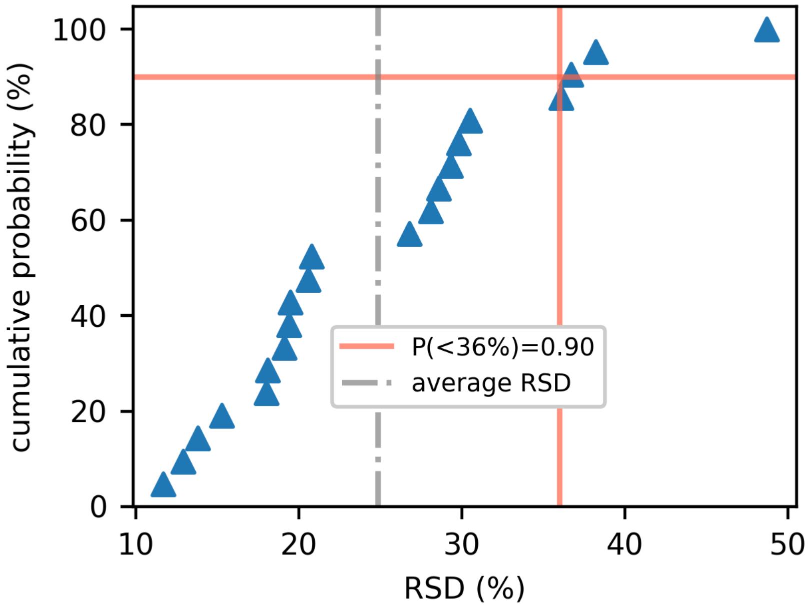

Figure S1: The cumulative probability as calculated for the relative standard deviation (RSD) of percent fentanyl within each batch. Note outliers have been removed for this calculation. The vertical grey line represents the average or 'typical' relative standard deviation for a batch. The intersecting orange lines highlight a 90% cumulative probability that the relative standard deviation (RSD) of fentanyl in an opioid mixture is less than 36%.

Table S2: Mean, interquartile range (IQR) and relative standard deviation (RSD) for fentanyl concentration for repeated spots of drug sub-samples. Measurements on the sample solutions of sub-samples were repeated $n = 5$ times for the first (A) and last(B) sample within each batch.

batch num	mean (w/w%)	IQR (w/w%)	RSD (%)
1A	12.5	0.9	4.4
1B	12.0	0.6	4.1
2A	14.1	0.2	1.1
2B	12.2	0.4	10.7
3A	17.8	0.6	3.4
3B	11.8	0.7	5.7
4A	19.7	0.2	54.3
4B	12.2	0.7	3.6
5A	3.2	0.0	2.1
5B	4.0	0.2	4.2
6A	12.7	0.3	2.2
6B	8.9	0.0	6.2
7A	9.2	0.3	2.4
7B	6.8	0.5	4.9
8A	11.8	0.2	3.9
8B	15.3	0.7	2.6
10A	9.0	0.2	3.1
10B	13.0	0.6	3.4
11A	20.9	0.8	3.2
11B	17.4	1.1	4.3
14A	23.8	2.0	4.9
14B	18.0	1.3	5.8
15A	4.8	0.2	5.1
15B	7.7	0.5	3.8
16A	17.2	0.1	1.4
16B	19.4	1.0	4.1
18A	48.1	0.5	4.6
18B	25.6	0.3	1.8
19A	14.4	0.8	4.8
19B	12.1	0.2	2.5
20A	4.1	0.1	2.9
20B	5.3	0.1	3.2
21A	12.4	0.3	2.1
21B	16.3	0.3	4.5
22A	37.2	0.4	2.7
22B	47.5	0.9	3.1
23A	23.4	0.7	2.5
23B	22.1	0.2	1.0
24A	7.5	0.6	8.6
24B	8.4	0.3	2.7
25A	13.6	0.4	6.4
25B	8.5	0.0	1.6
